# When mitochondria lose their fold: matrix proteostasis and stress signaling

**DOI:** 10.3389/fphys.2026.1873221

**Published:** 2026-06-15

**Authors:** Nils Bertram, Dejana Mokranjac

**Affiliations:** LMU Munich, Biozentrum – Cell Biology, Martinsried, Germany

**Keywords:** Hsp70, mitochondria, mitochondria-nuclear signaling, protein aggregation, proteostasis

## Abstract

A dedicated network of chaperones and proteases is present in the mitochondrial matrix that orchestrates import, folding, disaggregation and eventually degradation of proteins. When this network is overwhelmed, unfolded or misfolded proteins accumulate in different types of aggregates which may either support recovery of functional proteins, initiate spatial sequestration or drive toxic aggregation. Here, we discuss mitochondrial protein aggregation and how mitochondrial proteostasis stress is communicated to the rest of the cell.

## 1 Introduction

Proteostasis is the dynamic balance between protein synthesis, folding and degradation that allows cells to sustain a functional proteome (Sontag et al., 2017). To maintain this balance and safeguard the proteome from endogenous and exogenous perturbations, cells rely on a specialized network of proteins, collectively known as the protein quality control (PQC) network, that detects and either refold or degrade misfolded or damaged polypeptides (Sala et al., 2017; Hipp et al., 2019). Dysregulation or failure of the PQC network is a major contributing factor in numerous human diseases, highlighting the importance of this area of research. Proteostasis must be maintained not only in the cytosol, but also within the various organelles of the cell. In this context, mitochondria are of particular interest, not only because of their clinical implications but also because the majority of their proteome is hidden from the cytosolic PQC network by one or even two membranes. Mitochondria perform essential cellular functions ranging from ATP synthesis and formation of iron-sulfur clusters to initiation of apoptosis (Pfanner et al., 2019; Suomalainen and Nunnari, 2024). Reflecting their endosymbiotic origin, mitochondria retain a remnant of their ancestral genome and a complete machinery required for its expression (Gustafsson et al., 2016). However, the vast majority of mitochondrial proteins are encoded in the nucleus, synthesized on cytosolic ribosomes, and imported mostly post-translationally into the organelle in an unfolded state (Neupert, 2015; Hansen and Herrmann, 2019; Endo and Wiedemann, 2025). Because more than half of mitochondrial proteins are targeted to the organelle by N-terminal presequences and ultimately enter the matrix through the TOM and TIM23 complexes in the outer and inner membranes, respectively, mitochondrial proteostasis is tightly linked to import, folding, and proteolytic quality control in the matrix.

Cytosolic pathways that are activated in response to mitochondrial dysfunction have been extensively reviewed elsewhere and are not the focus of this mini-review (Song et al., 2021; Pfanner et al., 2025). Instead, we want to focus on matrix proteostasis, with particular emphasis on protein aggregation, and the possibility that mtHsp70 acts as an early sensor of mitochondrial stress.

### 1.1 Mitochondrial protein quality control network

**Figure 1:**
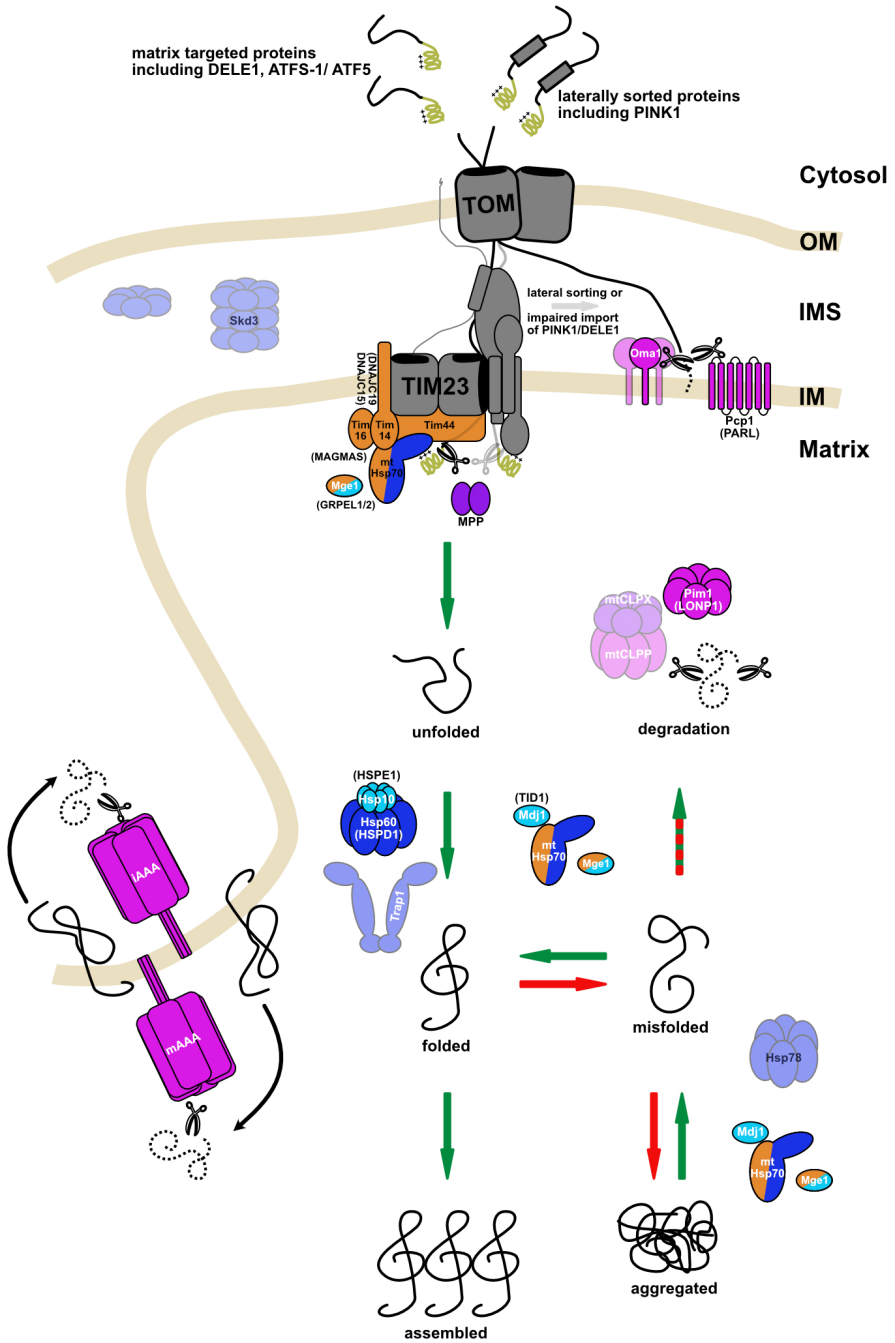
Mitochondrial proteostasis network. Schematic overview of mitochondrial import, protein folding, refolding, disaggregation, and degradation pathways. Main labels follow *Saccharomyces cerevisiae* nomenclature; human homologues are indicated in brackets, where applicable. Faded proteins denote factors that are absent from one of the two systems. Mitochondrial chaperones and their co-chaperones are shown in different shades of blue whereas mitochondrial proteases are shown in magenta. mtHsp70 system within the TIM23 complex involved in import of proteins from the cytosol is shown in orange. See text for details. OM, outer membrane, IM, inner membrane.

Mitochondria possess a dedicated mitochondrial protein quality control (mtPQC) network that is distinct from the cytosolic PQC but closely resembles the bacterial proteostasis machinery, in agreement with the endosymbiotic origin of the organelle (Pfanner et al., 2019; Deshwal et al., 2020; Voos and Pollecker, 2020). mtPQC network is highly conserved from yeast to humans and it comprises systems for folding, disaggregation, and proteolytic turnover, thereby determining whether misfolded proteins are rescued, degraded, or allowed to accumulate. The main mitochondrial import, folding, disaggregation, and degradation pathways discussed in this section are summarized in **Figure 1**. Mitochondria contain representatives of the three main chaperone families, Hsp60, Hsp70 and Hsp90 as well as their dedicated co-chaperones. The molecular mechanisms and ATP-hydrolysis driven cycles of these systems have been covered in several excellent reviews (Hayer-Hartl et al., 2016; Rosenzweig et al., 2019; Biebl and Buchner, 2019) and are therefore not discussed in detail here.

A central component of mtPQC network is the mitochondrial Hsp70 chaperone (mtHsp70). Together with the J domain protein (JDP) Mdj1 and the nucleotide exchange factor Mge1, mtHsp70 supports folding of newly imported proteins and prevents protein aggregation in the matrix (Kang et al., 1990; Rowley et al., 1994; Westermann et al., 1995), whereas together with another JDP Jac1 it contributes to biogenesis of iron-sulfur clusters (Craig and Marszalek, 2002). Importantly, about 10% of the mtHsp70 population (Morgenstern et al., 2017) is associated with the inner mitochondrial membrane, where it functions as part of the import motor of the TIM23 complex. There, mtHsp70 is recruited by Tim44, and its ATPase activity is controlled by JDP Tim14 (Pam18) and the J-like protein Tim16 (Pam16) (reviewed in Craig, 2018 and Mokranjac, 2020). An unusual property of mtHsp70 is its tendency to aggregate and mitochondria contain a specific chaperone Hep1/Tim15/Zim17 to maintain the functionality of mtHsp70 (Sanjuán Szklarz et al., 2005; Sichting et al., 2005).

mtHsp70 cooperates closely with the Hsp60/Hsp10 system, the mitochondrial counterpart of bacterial GroEL/GroES (Nisemblat et al., 2015; Hayer-Hartl et al., 2016). Mutations in Hsp10 and Hsp60 lead to increased aggregation of mitochondrial proteins, highlighting the importance of this system for initial folding and for maintaining mitochondrial protein homeostasis (Martin et al., 1992; Rospert et al., 1993). In higher eukaryotes, mitochondria also contain the Hsp90 homolog TRAP1 (Felts et al., 2000). Although the role of TRAP1 remains insufficiently understood, it has been implicated in cancer- and Alzheimer´s disease-linked mitochondrial dysfunction (Masgras et al., 2017; Dekker and Rüdiger, 2021). Consistent with a role in mitochondrial proteostasis, inhibition of TRAP1 induces mitochondrial protein-folding stress, remodeling of the Hsp60–Hsp10 chaperonin system, and activation of mitochondrial stress responses (Michaelis et al., 2022; Ehses et al., 2026).

When folding fails, mitochondria employ disaggregases and proteases to control their active proteome (Voos, 2013; Ruan et al., 2020b). In yeast, Hsp78, the mitochondrial homologue of bacterial ClpB, fulfills the role of the major disaggregase in the matrix and is indispensable for re-solubilization of protein aggregates after heat shock (von Janowsky et al., 2006). In cooperation with the mtHsp70–Mdj1 system, Hsp78 can resolubilize stress-induced protein aggregates, enabling either refolding or proteolytic clearance by matrix proteases (Wagner et al., 1994; Schmitt et al., 1995; Krzewska et al., 2001). Recent work suggests that Hsp78 does not simply promote substrate delivery to proteases, but rather acts in a triage pathway that can either protect stress-damaged proteins from premature degradation by facilitating their recovery or permit proteolytic clearance when refolding fails (Jaworek et al., 2022; Bertgen et al., 2024). In metazoans, protein disaggregation in the mitochondrial matrix is less well defined. Although a ClpB homolog SKD3/ClpB has been identified in human mitochondria, it was recently shown to localize to the intermembrane space, making its direct role in matrix proteostasis less likely (Weith et al., 2025). Instead, the mitochondrial mtHsp70–Mdj1 (human Mdj1 homologue is known as TID1) system has been proposed to play a central role in protein disaggregation in mammalian mitochondria (Iosefson et al., 2012).

Protein degradation within mitochondria is mediated by compartment-specific proteases rather than by the cytosolic ubiquitin–proteasome system (Deshwal et al., 2020). In the matrix, degradation of unstable or misfolded proteins is mainly carried out by the homologue of bacterial Lon protease known as Pim1 in yeast and LONP1 in mammals. This ATP-dependent protease functionally cooperates with mtHsp70 and Hsp60 to remove faulty proteins before they accumulate further (Wagner et al., 1994; Bender et al., 2011). Deletion of Pim1 in yeast leads to accumulation of lesions within the mitochondrial DNA and a loss of respiratory competence (Suzuki et al., 1994; van Dyck et al., 1998; Göke et al., 2020). Recently, a complex of Pim1, Mrx6 and Mam33 that regulates mtDNA copy number was identified in yeast (Göke et al., 2020; Schrott et al., 2026). However, although the general function of Lon appears to be conserved from yeast to humans (Matsushima et al., 2010; Zurita Rendón and Shoubridge, 2018), whether an analogous complex exists in mammals remains unclear as the homologue of Mrx6 has not yet been identified in mammalian cells. Mitochondrial inner membrane harbors two distinct AAA proteases, m-AAA and i-AAA proteases, that expose their catalytic sites to the matrix and the intermembrane space, respectively, surveying membrane proteins from the opposite sides of the inner membrane (Leonhard et al., 2000). In metazoans, ClpXP constitutes a second soluble matrix protease system that is absent in yeast (Deshwal et al., 2020). Current evidence suggests that its substrate spectrum is more restricted, positioning it as a selective, stress-responsive protease rather than a bona fide bulk degradation machinery (Nouri et al., 2020). Beyond housekeeping functions, mitochondrial proteases also participate in stress signaling, as illustrated by PARL-mediated processing of PINK1 under homeostatic conditions (Jin et al., 2010) and stress-induced cleavage of DELE1 by OMA1 (Fessler et al., 2020, 2022; Guo et al., 2020).

### 1.2 Protein aggregation in mitochondrial matrix

**Figure 2:**
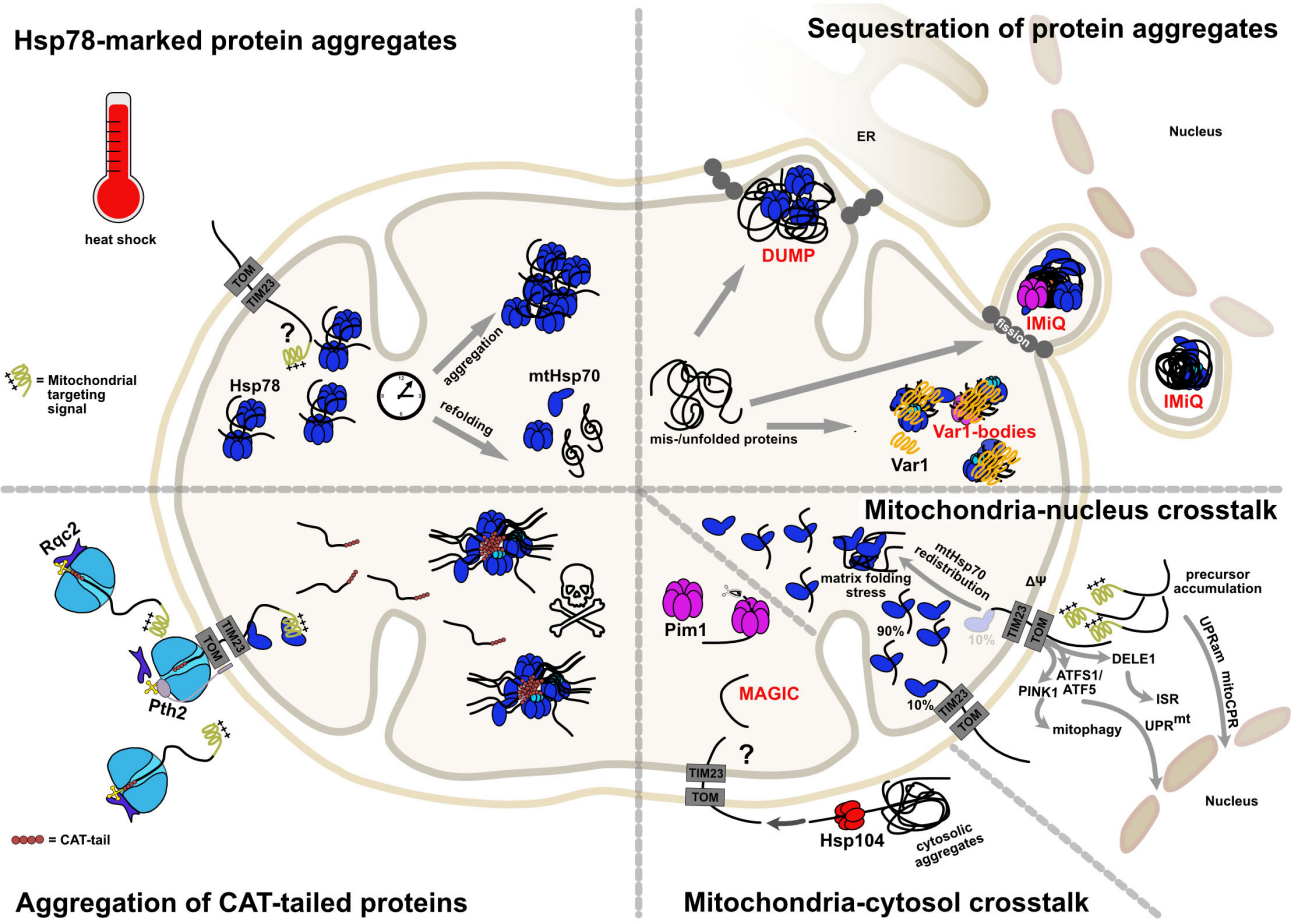
Mitochondrial aggregate classes and stress signaling during proteotoxic stress. Schematic overview of mitochondrial protein aggregation pathways and their cellular consequences. See text for details.

A failure of mitochondrial proteostasis, whether caused by acute stress or intrinsic defects, leads to the accumulation of unfolded proteins and their aggregation (Voos, 2013; Ruan et al., 2020b). As summarized in **Figure 2** and **Table 1**, these aggregates are not necessarily equivalent. Depending on the context, they may be transient structures associated with recovery, more stable sequestration sites that spatially confine proteotoxic material, or deposits that mark failed clearance. This distinction is central for interpreting the different aggregate classes described in yeast.

**Table 1:** Reported mitochondrial protein aggregates and their salient features.

Aggregate	Organism	Induction	Transient/persistent	Proteostasis components present	References
Hsp78-marked aggregates	*Saccharomyces cerevisiae*	Heat shock (fermentation)	Transient/Persistent	Hsp78 (fluorescence microscopy)	Vazquez‐Calvo et al., 2023; Bertgen et al., 2024
		Heat shock (respiration)	Transient		Vazquez‐Calvo et al., 2023
		Expression of misfolded proteins	Transient (mDHFR^mut^) Persistent (mtCPY*)		Bertgen et al., 2024
		Exposure to chemicals	Not analyzed		Vazquez‐Calvo et al., 2023
		ATP synthase assembly stress	Not analyzed		
		Cellular aging	Not analyzed		
		Mild oxidative stress	Not analyzed	Hsp78 (fluorescence microscopy and aggregate isolation)	Taskin et al., 2026
Var1-bodies	*Saccharomyces cerevisiae*	Heat shock	Persistent	Var1 (fluorescence microscopy); no colocalization with Hsp78	Bertgen et al., 2024
		Reduced mtHsp70 function	Not analyzed	Var1 (aggregate isolation)	
		Loss of Pim1 or Hsp78			
		Expression of misfolded proteins (mtCPY*)		Var1 (fluorescence microscopy and aggregate isolation)	
		Nuclear expression of Var1		Var1 (fluorescence microscopy) mtHsp70, Ssq1, Mdj1, Hsp60, Tcm62, Mcx1, Pim1, Yta10 and Yta12 (Var1 pulldown followed by MS)	
IMiQs	*Saccharomyces cerevisiae*	Heat shock	Persistent	Not analyzed	Bruderek et al., 2018
		Expression of misfolded proteins (mDHFR^mut^)		Hsp78, mtHsp70, Hsp60 (fluorescence microscopy) Mdj1, mtHsp70, Ecm10, Hsp60, Hsp78, Mcx1, Pim1, Cym1, Mas2/Mas1 (reporter pulldown followed by MS and/or immunodecoration)	Bruderek et al., 2018; Ruland et al., 2025
DUMPs	*Saccharomyces cerevisiae*	Cellular aging	Persistent	Hsp78 (fluorescence microscopy)	Ruan et al., 2020a; Sun et al., 2023
		Expression of misfolded proteins (mitoFluc)		Hsp78 (fluorescence microscopy) mtHsp70, Hsp10 (reporter pulldown followed by MS)	Ruan et al., 2020a
	Human cells (HEK293 and RPE1)	Expression of misfolded proteins (mitoFluc)		Not analyzed	
CAT-tailed protein aggregates	*Saccharomyces cerevisiae*	Non-stop mitochondrial reporter	Persistent	Hsp60, mtHsp70, Ssq1, Hsp78, Mdj1, Tcm62, Mcx1, Bcs1, Pim1 (reporter pulldown and aggregate isolation analyzed by MS)	Izawa et al., 2017; Bertram et al., 2025
		Loss of RQC components		mtHsp70, Ssq1, Hsp60 (aggregate isolation)	
	Human cells (HEK293, HeLa)	non-stop/poly-alanine reporter and loss of RQC or clearance components		ClpX (reporter pulldown); no interaction with Lonp1	Lv et al., 2024

#### 1.2.1 Hsp78-marked aggregates

Multiple perturbations, including heat shock, induce Hsp78-positive foci that can be observed within mitochondria by fluorescence microscopy. Under the same conditions, electron-dense aggregates were observed by electron microscopy (Vazquez‐Calvo et al., 2023; Bertgen et al., 2024). Consistent with a role in stress-dependent substrate binding, the interaction of ATPase-deficient Hsp78 with client proteins increases strongly after heat shock, and mtHsp70 is among the chaperones that are associated with Hsp78 clients and aggregates that appear after heat shock (Jaworek et al., 2022). This suggests that the Hsp78–mtHsp70 interaction may contribute to the re-establishment of mitochondrial homeostasis after stress. However, it remains unclear whether mtHsp70 is itself part of the pool of aggregated clients in this setting or whether it stimulates Hsp78 function, as known for the cytosolic disaggregation system (von Janowsky et al., 2006; Lee et al., 2013).

The formation and fate of the Hsp78-positive structures depend strongly on cellular context. In fermenting cells, acute heat stress induces several small Hsp78 foci that progressively coalesce, often into one larger structure that remains stable during recovery, whereas respiring cells form fewer aggregates and remove them more efficiently (Vazquez‐Calvo et al., 2023). After heat shock, Hsp78 co-localizes with both endogenous unstable proteins and unstable proteins artificially targeted to the matrix (Bertgen et al., 2024). Newly synthesized proteins are thought to be particularly susceptible to stress-induced misfolding, as their initial folding depends strongly on chaperone assistance and they encounter a markedly different environment immediately after import, which may further compromise folding efficiency (Voos, 2013; Ruan et al., 2020b). Whether newly imported precursor proteins constitute the principal aggregation-prone pool remains unresolved. One study reported that cycloheximide suppresses Hsp78-positive aggregate formation (Vazquez‐Calvo et al., 2023), whereas another observed no appreciable effect of cycloheximide treatment (Bertgen et al., 2024). This discrepancy may reflect differences in the severity of the heat stress applied in the respective experiments. Vázquez-Calvo et al. used a relatively mild heat shock (37 °C), whereas Bertgen et al. employed harsher conditions (40 °C). Thus, newly imported mitochondrial proteins may represent a particularly vulnerable source of aggregation under mild stress, while under more severe stress conditions, additional proteins are likely to contribute. This interpretation is consistent with the recent study showing that Hsp78 limits the aggregation of unprocessed mitochondrial precursors upon mild mitochondrial oxidative stress (Taskin et al., 2026). Together, these studies support a model in which Hsp78 acts as an early protective factor during acute mitochondrial proteotoxic stress and is associated mainly with transient, potentially recoverable aggregates. However, persistent Hsp78 bodies can also arise upon expression of an intrinsically misfolded matrix protein such as mtCPY*, and in that case expression is harmful for the cell. Hsp78-associated structures therefore do not appear to be per se beneficial or harmful, but rather mark mitochondria under substantial proteotoxic stress (Bertgen et al., 2024).

#### 1.2.2 Var1-bodies

A second and more persistent class of protein aggregates described in the matrix of yeast mitochondria is nucleated by Var1, the mitochondrially-encoded protein of the small ribosomal subunit also known as uS3m (Greber and Ban, 2016). Unlike other S3-family proteins, mitochondrially-encoded Var1 is exceptionally rich in asparagine residues and has a pronounced intrinsic tendency to aggregate, particularly through mitochondria-specific loop regions (Bertgen et al., 2024). Under non-stressed conditions, Var1 is kept soluble by the mitochondrial chaperones and is incorporated into the small mitoribosomal subunit. Under acute proteotoxic stress, however, Var1 undergoes a transition from a soluble, chaperone-stabilized protein to an insoluble, aggregated state, giving rise to punctate structures termed Var1-bodies. Var1 aggregation is promoted not only by heat shock, but also by impaired mtHsp70 function, loss of Pim1, loss of Hsp78, or expression of mtCPY*, indicating that it responds broadly to mitochondrial folding stress rather than to one particular perturbation. Notably, Var1-bodies do not appear to function simply as passive end accumulations. A nuclear-encoded version of Var1 retained its aggregation-prone behavior and was sufficient to protect cells against acute heat stress even when mitochondrial translation was blocked, indicating that the protective effect of Var1 can be uncoupled from its canonical role in the mitoribosome. Proteomic analyses further showed that Var1 expression suppresses aggregation of many mitochondrial proteins during stress. Together, these observations support a model in which Var1-bodies act as sequestration sites that absorb misfolded or metastable matrix proteins during acute stress and thereby protect the remaining mitochondrial proteome (Bertgen et al., 2024). Although the uS3m/S3 protein family is conserved in mammals, the aggregation-prone, asparagine-rich loop regions in yeast Var1 are absent from its human counterpart. It will be interesting to determine whether an analogous nucleating mechanism exists in human mitochondria.

#### 1.2.3 Intramitochondrial protein quality control compartments (IMiQ) and deposits of unfolded mitochondrial proteins (DUMP)

When the mtPQC capacity is exceeded, misfolded proteins in mitochondria can also partition into more persistent inclusions. In yeast, the intramitochondrial protein quality control compartment (IMiQ) was defined as a dedicated deposit site for misfolded matrix proteins (Bruderek et al., 2018). Aggregation-prone reporters do not remain diffusely distributed throughout the mitochondrial network, but instead accumulate in one or several electron-dense, amorphous structures that often localize close to but inside the nucleus (Bruderek et al., 2018; Ruland et al., 2025). Importantly, this sequestration markedly limits proteotoxicity: despite near-complete aggregation of a reporter protein, only minor amounts of endogenous mitochondrial proteins co-aggregate, whereas the remaining mitochondrial network largely preserves membrane potential, import competence, respiratory complex organization, and growth capacity. IMiQ deposits are also retained in the mother cell during budding, indicating that spatial sequestration not only neutralizes damaged proteins within mitochondria but also restricts their transfer to daughter cells (Bruderek et al., 2018).

IMiQ formation is an active process that depends on mitochondrial dynamics and local PQC activity. Fusion-deficient cells fail to concentrate aggregates into a single IMiQ compartment and instead accumulate many smaller inclusions, whereas fission-deficient cells form fewer but larger aggregates that remain connected to the mitochondrial network and can become proteotoxic (Ruland et al., 2025). Chaperone association with mitochondrial aggregates appears stage- and context-dependent. mtHsp70 and Hsp78 can both associate with initial mitochondrial aggregates (Bruderek et al., 2018; Ruland et al., 2025), although the recruitment may be transient or restricted to earlier stages. Mature IMiQ deposits were reported to lack detectable mtHsp70 in microscopy and only showed minor enrichment of mtHsp70 and Hsp60 in biochemical experiments (Bruderek et al., 2018). These observations suggest that sequestration is not a passive precipitation, but part of an organized response in which chaperones and mitochondrial dynamics shape the aggregate state.

A similar intramitochondrial deposit was described as the deposit of unfolded mitochondrial proteins (DUMP) (Ruan et al., 2020a). Excessive unfolded matrix proteins are consolidated into detergent-resistant, FRAP-stable, solid-phase inclusions within mitochondria. DUMP formation increases with cellular age, is accelerated by exogenous unfolded proteins, and occurs at mitochondria-ER contact sites, with a requirement for Ups1 and Tam41. DUMP-containing cells exhibit reduced membrane potential, impaired growth, mtDNA instability, and shortened replicative lifespan, but also promoted the asymmetric retention of damaged mitochondrial material in mother cells (Sun et al., 2023). DUMP and IMiQ therefore share several key features: both were identified by expression of aggregation-prone proteins in the matrix, both represent intramitochondrial deposits enclosed by mitochondrial membranes, and both are linked to asymmetric segregation during cell division. DUMP is associated with more overt dysfunction and with mitochondria-ER contact sites, whereas IMiQ was emphasized as a more protective form of sequestration. These differences may reflect distinct aggregate burdens or different stages of a related sequestration process rather than completely unrelated entities, although this relationship remains inferential rather than established. However, consistent with this idea, perturbing mitochondrial dynamics alters IMiQ maturation: loss of fusion impairs aggregate coalescence, whereas loss of fission impairs physical separation from the mitochondrial network, conditions that can reduce cellular fitness under stress (Ruland et al., 2025). Interestingly, DUMP-like structures were also identified in human cells, suggesting that related matrix-sequestration principles may be conserved (Ruan et al., 2020a).

#### 1.2.4 Toxic aggregation of CAT-tailed proteins

The aggregate classes discussed above are often interpreted as protective, adaptive, or at least context-dependent responses to mitochondrial proteotoxic stress. However, not all aggregation states can be understood in this way. Some protein aggregation events are clearly toxic and associated with mitochondrial dysfunction (Vijayvergiya et al., 2005; Izawa et al., 2017; Ehses et al., 2026).

A well characterized example in yeast is the mitochondrial aggregation of CAT-tailed proteins. CAT-tailed proteins arise from the aberrant translation products and are generated by the ribosomal quality control (RQC) pathway (Izawa et al., 2017; Lv et al., 2024; Bertram et al., 2025). RQC is triggered by ribosomal collisions induced by translational stalling, leading to dissociation of the ribosome into its 40S and 60S subunits (reviewed in Joazeiro, 2019). The 60S subunit remains tethered to the nascent chain through the tRNA in the P site, and release of this stalled nascent chain requires a coordinated action of Vms1, Ltn1, and Rqc2, followed by ubiquitination and proteasomal degradation. The process also involves the non-canonical C-terminal addition of alanine and threonine residues, termed CAT tailing. When this pathway fails, nuclear-encoded mitochondrial proteins bearing CAT tails are released by the peptidyl-tRNA hydrolase Pth2 and are imported into mitochondria where they promote protein aggregation (Izawa et al., 2017; Bertram et al., 2025). Isolation of these aggregates followed by mass spectrometry showed that they sequester not only components of the mitochondrial proteostasis network, but also key factors involved in respiration and mitochondrial translation (Izawa et al., 2017). Consistent with this, failure of RQC can lead to mitochondrial dysfunction and, under conditions of sustained proteotoxic stress, loss of viability or cell death (Heo et al., 2010; Izawa et al., 2017; Geng et al., 2024; Lv et al., 2024; Bertram et al., 2025). Even in the absence of a global RQC defect, mitochondrial targeting of artificially CAT-tailed GFP is sufficient to induce severe growth defects and visible aggregation (Bertram et al., 2025). Thus, in contrast to the aggregate types discussed above, protein aggregates linked to failed mitoRQC provide a clear example of toxic protein accumulation driven by defective cytosolic surveillance before or during mitochondrial protein biogenesis.

#### 1.2.5 The MAGIC pathway

An intriguing extension of mitochondrial proteostasis is the possibility that mitochondria can buffer cytosolic proteotoxic stress (**Figure 2**). Cytosolic aggregates formed during heat shock were reported to associate with mitochondria and to interact with the TOM complex (Ruan et al., 2017). Subsequent work further implicated Tom70 and nascent mitochondrial precursor proteins in the localized condensation of cytosolic protein aggregates on the mitochondrial surface (Liu et al., 2023). Their clearance required not only the cytosolic disaggregase Hsp104, but also an intact mitochondrial membrane potential and the presequence import pathway, as shown using temperature-sensitive mutants (Ruan et al., 2017). Split-GFP and APEX-based approaches further suggested that highly aggregation-prone cytosolic proteins can enter the mitochondrial matrix where they are degraded by Pim1. This pathway, termed MAGIC (“mitochondria as guardian in cytosol”), appears to be metabolically regulated by Snf1/AMPK. Under high-glucose conditions, import of misfolded proteins into mitochondria is favored, whereas glucose limitation activates Snf1/AMPK, suppressing MAGIC to prioritize mitochondrial biogenesis and respiratory fitness (Wang et al., 2024). MAGIC therefore does not appear to be a constitutive disposal route, but rather a context-dependent and potentially moonlighting function of mitochondria. Excessive mitochondrial accumulation of misfolded cytosolic proteins can impair yeast growth and reduce mitochondrial membrane potential (Wang et al., 2024). Thus, even if MAGIC can buffer cytosolic proteotoxicity, it may do so at a cost to the organelle itself. The physiological significance and especially the mechanism by which proteins lacking canonical mitochondrial targeting signals enter the matrix, remain unresolved.

### 1.3 mtHsp70 as a sensor of mitochondrial stress

A central question is how mitochondrial dysfunction is sensed and this information relayed to the cytosol/nucleus to elicit rescue/degradation programs. In mammalian systems, several proteins have been identified, including DELE1, PINK1, and ATF5, whose defective import into mitochondria serves as a signal of mitochondrial stress (**Figure 2**). Under homeostatic conditions, DELE1 is continuously imported and degraded within the mitochondrial matrix, whereas under stress conditions, its import is stalled at the level of the inner membrane, leading to its cleavage by OMA1 and release to the cytosol, where it activates the integrated stress response (Fessler et al., 2020; Guo et al., 2020). Similarly, impaired import of PINK1 results in its accumulation at the outer mitochondrial membrane and activation of mitophagy (Jin et al., 2010). In *C. elegans*, the transcription factor ATFS-1 is normally imported into mitochondria and degraded, but upon import failure it is redirected to the nucleus, where it activates the UPRmt (Nargund et al., 2012; Rolland et al., 2019). Whereas the mammalian transcription factor ATF5 appears to function analogously (Fiorese et al., 2016), in yeast, no direct mitochondrial stress sensor has yet been identified; however, accumulation of non-imported mitochondrial precursor proteins in the cytosol activates proteostatic response pathways such as mPOS and mitoCPR (Wang and Chen, 2015; Wrobel et al., 2015; Weidberg and Amon, 2018). Together, these observations identify mitochondrial protein import as a critical bottleneck that can serve as a sensitive readout of mitochondrial function.

Because most presequence-containing matrix proteins are imported through the TOM and TIM23 complexes in a membrane potential-dependent manner (Neupert, 2015; Hansen and Herrmann, 2019; Endo and Wiedemann, 2025) import failure has often been interpreted primarily as a consequence of membrane depolarization. However, these quality control pathways can also be activated in mitochondria with intact membrane potential, indicating that complete membrane depolarization is unlikely to be the major, physiologically-relevant signal that initiates stress responses (Jin and Youle, 2013; Poveda-Huertes et al., 2020; Michaelis et al., 2022; Banerjee et al., 2026). In particular, accumulation of misfolded proteins in the matrix was shown to stabilize PINK1 and recruit PARKIN to polarized mitochondria (Jin and Youle, 2013). Furthermore, impaired import motor function and aggregation of import motor components that was induced by proteostasis failure can reduce mitochondrial protein import prior to the collapse of the membrane potential (Jin and Youle, 2013; Michaelis et al., 2022). These findings suggest that proteostasis defects can compromise mitochondrial quality control and protein import before global mitochondrial failure becomes apparent.

In this context, the dual role of mtHsp70 becomes relevant. mtHsp70 is both a core chaperone in the mitochondrial matrix and an essential component of the import motor of the TIM23 complex. Using single-molecule FRET, a recent study showed that mtHsp70 is predominantly present in the substrate-bound state in physiologically active mitochondria, suggesting that the mtHsp70 network operates near the limit of its capacity (Banerjee et al., 2026). Importantly, only a minor fraction of mtHsp70, approximately 10%, localizes to the import sites (Morgenstern et al., 2017). Upon expression of an unfolded protein in the matrix, mtHsp70 dissociated from the TIM23 complex and was redirected toward folding or stabilization of the artificial substrate, even under conditions in which neither membrane potential loss nor pronounced protein aggregation could be detected. This removal of mtHsp70 from the import sites led to a specific reduction in mtHsp70-dependent import of proteins, whereas mtHsp70-independent import remained unaffected (Banerjee et al., 2026). Consistent with a limiting mtHsp70 pool, mtHsp70 is also recruited to different mitochondrial aggregates, where it is among the most prominent aggregate-associated chaperones (Jaworek et al., 2022; Michaelis et al., 2022; Bertram et al., 2025; Ruland et al., 2025). These findings suggest that mtHsp70 acts as an early sensor of mitochondrial proteotoxic stress by directly coupling matrix (re-) folding demand to import capacity. Protein import therefore appears not to be merely a downstream casualty of mitochondrial dysfunction, but one of the earliest processes to register declining matrix proteostasis. In this way, mtHsp70 emerges as a plausible molecular link between intramitochondrial proteotoxic stress and the mitochondria-nuclear signaling pathways that coordinate the cellular stress response. It is also reasonable to assume that mitochondrial stress sensing may be both tissue- and threshold-dependent. Although PINK1 and DELE1 are mostly broadly expressed, PARKIN, the canonical effector of PINK1-dependent mitophagy, is particularly enriched in tissues with high demand for mitochondrial respiration (brain, testis and kidneys) (The Human Protein Atlas, n.d). In contrast, ATF5 displays a more tissue-biased expression pattern and is particularly enriched in the liver, where mitochondria operate under heightened metabolic and proteotoxic stress (Mansouri et al., 2018; The Human Protein Atlas, n.d). Different cell types may therefore differ in how much mitochondrial proteotoxic stress they can tolerate and which stress-response pathway they preferentially engage. Some cell types may tolerate partially functional mitochondria and just remove completely nonfunctional ones, whereas others require tight regulation of mitochondrial function and are sensitive to already minor loss of function. Alternatively, these pathways may reflect different stages of mitochondrial damage, with DELE1- and ATF5-associated responses acting at earlier, potentially reversible stages, whereas PINK1–PARKIN signaling becomes dominant once damage exceeds a repairable threshold and mitophagy is required. In this context, the degree to which mtHsp70 needs to be titrated away from the TIM23 complex to trigger stress signaling may vary depending on the cell type, import load, mitochondrial proteostasis capacity and the actual protein whose import defect serves as a signal of mitochondrial dysfunction.

## 2 Outlook

Viewed together, these studies suggest that mitochondrial matrix proteostasis is not defined solely by the canonical activities of chaperones, disaggregases, and proteases, but also by how failing or overloaded PQC is reorganized to result in distinct aggregate and signaling states. Some aggregation events appear transient and compatible with recovery, others support spatial sequestration and asymmetric retention, and still others are directly toxic because they trap essential components of respiration, translation, or proteostasis networks. An important next step will be to understand how mitochondria channel misfolded proteins into refolding-, degradation-, sequestration- or toxic accumulation pathways. It will also be important to analyze whether the aggregates described in different experimental systems represent distinct entities or context-dependent variants of a common response. Equally important will be to determine how early proteostasis defects are communicated to the rest of the cell. In this regard, mtHsp70 is particularly compelling because it is situated at the intersection of matrix folding and protein import. This places mtHsp70 in a strong position to convert local proteostasis imbalance into altered import behavior and thereby into a signal that can engage broader mito-nuclear stress response pathways.
